# Transcutaneous electromyographic respiratory muscle recordings to quantify patient–ventilator interaction in mechanically ventilated children

**DOI:** 10.1186/s13613-018-0359-9

**Published:** 2018-01-24

**Authors:** Alette A. Koopman, Robert G. T. Blokpoel, Leo A. van Eykern, Frans H. C. de Jongh, Johannes G. M. Burgerhof, Martin C. J. Kneyber

**Affiliations:** 1 Division of Paediatric Intensive Care, Department of Paediatrics, Beatrix Children’s Hospital, University Medical Center Groningen, The University of Groningen, Internal Postal Code CA 62, P.O. Box 30.001, 9700 RB Groningen, The Netherlands; 2 Inbiolab B.V., Groningen, The Netherlands; 3 Faculty of Science and Technology, University of Twente, Enschede, The Netherlands; 4 Department of Epidemiology, University Medical Center Groningen, The University of Groningen, Groningen, The Netherlands; 5 Division of Paediatric Intensive Care, Department of Paediatrics, VU University Medical Center, Amsterdam, The Netherlands; 6 Critical Care, Anesthesia, Peri-operative Medicine and Emergency Medicine (CAPE), The University of Groningen, Groningen, The Netherlands

**Keywords:** Child, Mechanical ventilation, Asynchrony, Electromyography, Patient–ventilator interaction, Paediatric intensive care

## Abstract

**Background:**

To explore the feasibility of transcutaneous electromyographic respiratory muscle recordings to automatically quantify the synchronicity of patient–ventilator interaction in the pediatric intensive care unit.

**Methods:**

Prospective observational study in a tertiary paediatric intensive care unit in an university hospital. Spontaneous breathing mechanically ventilated children < 18 years of age were eligible for inclusion. Patients underwent a 5-min continuous recording of ventilator pressure waveforms and transcutaneous electromyographic signal of the diaphragm. To evaluate patient–ventilator interaction, the obtained neural inspiration and ventilator pressurization timings were used to calculate trigger and cycle-off errors of each breath. Calculated errors were displayed in the dEMG-phase scale.

**Results:**

Data of 23 patients were used for analysis. Based on the dEMG-phase scale, the median rates of synchronous, dyssynchronous and asynchronous breaths as classified by the automated analysis were 12.2% (1.9–33.8), 47.5% (36.3–63.1), and 28.9% (6.6–49.0).

**Conclusions:**

The dEMG-phase scale quantifying patient–ventilator breath synchronicity was demonstrated to be feasible and a reliable scale for mechanically ventilated children, reflected by high intra-class correlation coefficients. As this non-invasive tool is not restricted to a type of ventilator, it could easily be clinical implemented in the ventilated pediatric population. However; correlation studies between the EMG signal measured by surface EMG and esophageal catheters have to be performed.

## Background

Patient–ventilator asynchrony (PVA) in mechanically ventilated adults is associated with prolonged duration of mechanical ventilation (MV), increased use of sedatives and longer intensive care unit (ICU) and hospital stay [1–3]. Although the occurrence of PVA in mechanically ventilated children is common as we and others have shown, the relationship between PVA and clinical outcome is unclear for this group of patients [4–6].

Previously, we have shown in a heterogeneous group of mechanically ventilated children that one out of every three breaths was out of sync when the airway pressure and flow waveforms were visually inspected [4]. However, such inspection is cumbersome and may not reflect the true prevalence of PVA as the neural breathing drive is not taken into consideration. Alternatively, electrical activity of the diaphragm measured with a specific nasogastric catheter (EAdi) or the esophageal pressure signal can be used and is in fact more accurate signals for identifying PVA [6–11].

So far, use of these methods has been restricted to research purposes mainly because of the lack of ability to provide the clinician with real-time feedback of the level of PVA. In order to truly understand the clinical relevance of PVA in mechanically ventilated children, there needs to be a system that provides such feedback on both the occurrence and type of PVA. Recent advances have been made in the development of tools to automatically identify PVA [2, 12–15]. Such real-time automatic analyses are needed for clinical trials investigating the efficacy of interventions targeted at reducing PVA and on patient outcome. Sinderby et al. [16] developed an automated, objective and standardized neural index to quantify patient–ventilator interaction (NeuroSync) based on the measurements of EAdi and ventilator pressure waveforms. Determining patient–ventilator interaction by this method had a higher inter-rater reliability and proved to be more sensitive than manual analysis.

However, this new approach is only limited to ventilators capable of measuring EAdi. Furthermore, it mandates the insertion of an esophageal catheter which may be a disadvantage especially in the pediatric context. Transcutaneous recording of the electromyographic signals of the diaphragm (dEMG) may be considered as a suitable alternative [17–19]. Although at this moment no correlation studies between dEMG and EADi have been performed, this non-invasive, easy-to-perform technique provides reproducible electromyographic signals of the diaphragm [17]. We therefore tested the hypothesis that it would be feasible to automatically detect, quantify and display patient–ventilator interactions using a modified NeuroSync index (dEMG-phase scale) in mechanically ventilated children when analyzing dEMG together with ventilator pressure and flow versus time waveform.

## Methods

### Study population

This study was performed at the pediatric intensive care unit (PICU) of the Beatrix Children’s Hospital, University Medical Center Groningen between February and July 2015. The Institutional Review Board approved the study. Signed informed consent was obtained from both parents or legal caretakers. Mechanically ventilated children < 18 years of age were eligible for inclusion. Patients with congenital or acquired neuromuscular disorders, premature birth with gestational age corrected for post-conceptional age < 40 weeks, severe traumatic brain injury (i.e. Glasgow Coma Scale < 8), congenital or acquired damage to the phrenic nerve, congenital or acquired paralysis of the diaphragm, use of neuromuscular blockade, chronic lung disease (i.e. tracheostomy ventilation), severe pulmonary hypertension, contra-indication for placement of electrodes on the skin and patients unable to trigger the ventilator from any other cause were excluded.

### Study procedure

During the study, all patients remained subjected to standard-of-care of the intensive care. Measurements took place within the 24 h prior to extubation. The attending physician defined the ventilator mode and settings in agreement with our local guideline. Expiratory tidal volume (*V*_T_) was targeted at 6–8 mL/kg actual bodyweight. The flow trigger was set at 1.0 L/min. A proximal flow-sensor was used in patients < 15 kg. In cases of decreased respiratory system compliance, permissive hypercapnia was applied (pH > 7.20). The level of pressure support ventilation (PSV) was routinely set as PSV = peak inspiratory pressure (PIP) minus positive end-expiratory pressure (PEEP). Ventilator settings were fixed during the measurement unless the clinical condition of a patient required an adjustment of the setting made by the attending physician. Patients were ventilated in a time-cycled, pressure-limited synchronized mode of ventilation with PSV, pressure controlled/synchronized intermittent mandatory ventilation (PC/SIMV + PSV), pressure-limited mode with preset tidal volume (V_T_), i.e. pressure regulated volume controlled with PSV (PRVC/SIMV + PSV) or pressure controlled assist control (PC/AC) using the AVEA ventilator (CareFusion, Yorba Linda, CA, USA). Continuous infusion of midazolam, oral lorazepam and morphine or fentanyl intravenously was given for analgesia–sedation. The COMFORT behavior scale was used to titrate the level of sedation [20, 21]. Ten minutes prior to the recordings, patients were suctioned and the circuit was cleared from any water. Patients were in a 30 degrees anti-Trendelenburg supine position.

### Ventilator pressure waveforms and dEMG acquisition

Patients underwent a 5-min continuous recording of ventilator pressure waveforms and dEMG. Ventilator pressure tracings were acquired through the ventilator’s RS232 interface (Ventilator Open XML Protocol, VOXP) at a sampling frequency of 100 Hz. The dEMG was derived from one pair of single Ag/AGCl electrodes (EasyTrode TM Pre gelled Electrodes, Multi Bio Sensors Inc, El Paso, USA) bilaterally placed at the costo-abdominal margin in the nipple line. A common electrode was placed at the sternal level [17]. The dEMG was recorded at a sampling frequency of 500 Hz using the Dipha (Inbiolab, Groningen, The Netherlands). Polybench software (Applied Biosignals GmbH, Weener, Germany) was used to record the pre-processed data from the ventilator and the EMG recording device. The ventilator pressure waveforms and electrical activity of the diaphragm were analyzed offline.

### Data processing

The recorded dEMG needed to be processed for reliable assessment of the respiratory neural drive. The electrical activity of the heart and other peak artifacts were isolated from the raw dEMG data by means of an extended version of the gating technique [22]. The gates were filled with the running average of the processed dEMG signal. A 50 Hz notch filter was used to minimize electrical interference from electronic devices on the intensive care. After filtering and gating, the running root mean square (RMS) (time window *T* = 0.2 s) of the processed dEMG signal was calculated. The calculated dEMG was used for analysis.

### Description of patient–ventilator interaction

To evaluate patient–ventilator interaction, the computed dEMG activity was both manually and automatically compared to the ventilator’s waveforms to calculate the dEMG-phase scale (dEMG-phase scale_MANU_ and dEMG-phase scale_AUTO_, respectively), using the modified NeuroSync method previously described by Sinderby et al. [16]. Two investigators (AK and RB) manually analyzed the ventilator pressure and dEMG tracings using a graphical interface designed in Polybench (Applied Biosignals GmbH, Weener, Germany). Each investigator individually placed markers in the interface at the onset of neural inspiration (NA_ON_), at 1/3 decline in the dEMG from its peak, i.e. the termination of neural inspiration (NA_OFF_), at the beginning of ventilator pressurization (MV_ON_) and at the end of ventilator pressurization (MV_OFF_). The obtained neural inspiration and ventilator pressurization timings: NA_ON_, NA_OFF_, MV_ON_ and MV_OFF_ were used to calculate trigger and cycle-off errors of each breath. The algorithm for automated analysis was designed according to the same rules as for manual analysis. Early trigger and cycle-off errors as well as late trigger and cycle-off errors could range between 0 and 100%. Limits whether a breath is synchronous, dyssynchronous were set, accordingly to Sinderby et al. [16], at ± 33% difference between NA_ON_ and MV_ON_ and NA_OFF_ and MV_OFF_. Neural inspirations not related to ventilator pressurizations or vice versa were considered as asynchronous breaths and assigned 100%. Cases of asynchronous breaths included ventilatory pressurization without neural activity (MV without NA), neural activity without ventilatory pressurization (NA without MV), multiple ventilatory pressurizations with one neural activity (multiple MV with NA) and multiple neural activities within one ventilatory pressurization (multiple NA with MV). Obtained data are shown in a graphical representation of the dEMG-phase scale; the intra-breath patient–ventilator interaction diagram. The dEMG-phase scale was defined as the mean absolute error of all breaths. The dEMG-phase scale_MANU_, which was obtained by both experts, was compared with the dEMG-phase scale_AUTO_.

### Baseline characteristics

Patient baseline demographics included age, gender, weight, admission diagnosis, Pediatric Index of Mortality (PIM) II and 24-h Pediatric RISk of Mortality (PRISM) II score, time of recordings and admission diagnosis. Before initiation of the measurements, ventilator settings including mode, pressure above peep (PAP), PEEP, mean airway pressure (*P*_mean_), PSV, expiratory tidal volume (*V*_T_), frequency of set breaths, fraction of inspired oxygen (FiO_2_) and inspiratory time were recorded. Clinical data included prior use of neuromuscular blockade, tube size, air leakage around the endotracheal tube (ETT), end tidal CO_2_ and received amount of analgesia-sedation in the last 4 h preceding the registration. The COMFORT score was evaluated during the recording [20, 21].

### Statistical analysis

The Shapiro–Wilk test was used to test data for normal distribution. Descriptive data were expressed as median [first quartile; third quartile] or percentage (%) of total. The breath-by-breath inter-rater agreement, defined as the agreement between errors obtained by the two investigators, and inter-method agreement, defined as the agreement between errors obtained by automated analysis and the average errors obtained by the two investigators, were evaluated by means of the intra-class correlation coefficient (ICC). Reliability was considered to be acceptable if the ICC was greater than 0.75 and excellent if the ICC was greater than 0.90 [23, 24]. After confirmation of a good breath-by-breath inter-rater and inter-method reproducibility, the agreement between dEMG-phase scale_MANU_ and dEMG-phase scale_AUTO_ was evaluated. All statistical analyses were performed using SPSS version 24 (IBM, Armonk, USA).

## Results

### Study population

Patient characteristics are summarized in Table 1. At the time of analysis, one patient was excluded because she developed meningitis with severe neurologic impairment on the measurement day. Thus, data of *N* = 23 (17 boys and 6 girls) patients were used for analysis. Seventeen (74%) patients were ventilated for respiratory failure of any cause; five patients (22%) were admitted after corrective cardiac surgery for congenital heart disease. One patient was ventilated for circulatory failure (4%). Median percentage of patient triggered breaths was 96% [61; 99]. At the time of data recording patients received a median morphine dosage of 10 mcg/kg/h [7.7; 10.5], median midazolam dosage of 0.1 mg/kg/h [0.08; 0.2] and median fentanyl dosage of 2.0 mcg/kg/h [1.0; 2.9]. One patient received lorazepam in a dosage of 0.3 mg/kg/day. Median COMFORT score was 14 [11, 15].

**Table 1:** Baseline demographics, mode of ventilation and ventilator settings

Variable	
*N*	23
Gender (male, *n*)	17
Pulmonary diagnosis (*n*)	17
Surgical diagnosis (*n*)	5
PIM II	− 2.9 [− 3.3; − 2.5]
PRISM II	11.0 [9.0; 15.0]
Age (months)	3.6 [1.4; 9.8]
Duration MV (days)	4.7 [2.9; 7.0]
Cuffed ETT (*n*)	14
Air leakage uncuffed ETT (%)	2.0 [0.0; 8.0]
End tidal pCO_2_ (kPa)	6.1 [5.7; 6.5]
COMFORT scale	14 [11; 15]
Expiratory *V*_T_/kg (ml)	7.2 [6.1; 8.3]
Patient triggered breaths (%)	96 [61; 99]
PC/AC (*N*)	21
PC/SIMV + PSV (*N*)	2
PAP (cm H_2_O)	13 [12; 14]
PEEP (cm H_2_O)	6 [5; 6]
Set frequency (/min)	25 [20; 30]
FiO_2_	0.35 [0.25; 0.40]
Inspiratory time (s)	0.55 [0.50; 0.65]
PSV (cm H_2_O)	13 [13]

*PIM II*, Pediatric Index of Mortality score II; PRISM II, Pediatric Risk of Mortality score II; MV, mechanical ventilation; ETT, endotracheal tube; End tidal pCO_2_, end tidal pCO_2_ before starting measurement; COMFORT scale, measurement tool to assess distress, sedation and pain in nonverbal paediatric patients; Expiratory *V*_T_/kg, expiratory tidal volume per kilogram body weight; PC/AC, pressure control/assist control; PC/SIMV + SIMV, pressure control/synchronized intermittent mandatory ventilation plus pressure support ventilation; PIP, pressure above PEEP; PEEP, positive end-expiratory pressure; FiO_2_, fraction of inspired oxygen; PSV, pressure support ventilation

### Description of dEMG recordings

The recorded dEMG showed the following characteristics. Median neural inspiration was 0.86 s [0.74; 1.0], neural expiration was 0.79 s [0.57; 0.96], median baseline dEMG signal was 1.16 μV [0.76; 2.12], peak dEMG signal was 2.69 μV [1.72; 3.62], and median amplitude was 1.06 μV [0.85; 1.87]. Two patients had no detectable dEMG signal, probably caused by sedation. For all other patients during the entire breathing cycle, no loss of the dEMG signal was observed. Median trigger error (i.e. dEMG signal compared to ventilator pressurization) for premature triggering was 0.33 s [0.24–0.46] and for delayed triggering 0.16 s [0.03–0.24]. Median cycle-off error for premature cycling was 0.11 s [0.07–0.20] and for delayed cycling 0.13 s [0.08–0.39].

### dEMG-phase scale_AUTO_

**Fig. 1:**
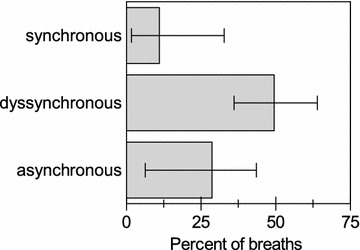
Rates of synchronous, dyssynchronous and asynchronous breaths as classified by the automated analysis. Columns are median, and bars are interquartile range

**Fig. 2:**
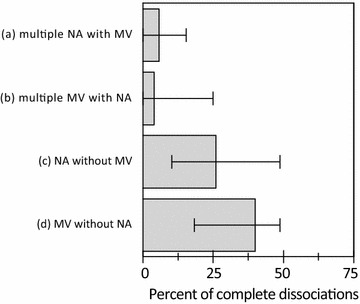
Rates of multiple NA with MV (a), multiple MV with NA (b), NA without MV (c) and MV without NA (d) as classified by the automated analysis. Columns are median, and bars are interquartile range

**Fig. 3:**
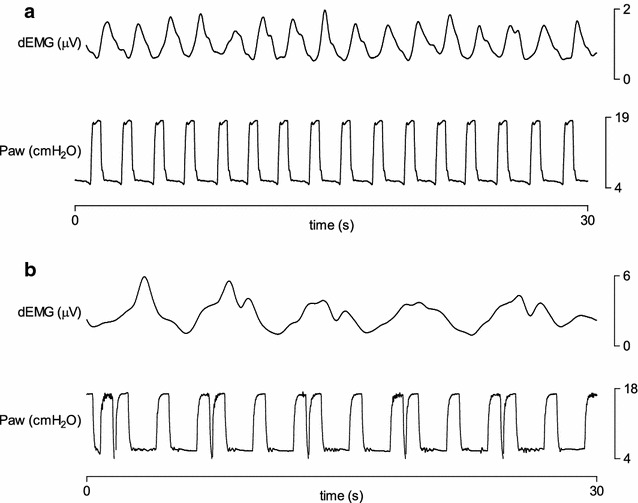
Representative examples of transcutaneous diaphragm EMG and ventilator pressure–time tracings. Patient A is showing good patient–ventilator interaction. Patient B is showing poor patient–ventilator interaction with multiple ventilator pressurization within one period of neural inspiration

**Fig. 4:**
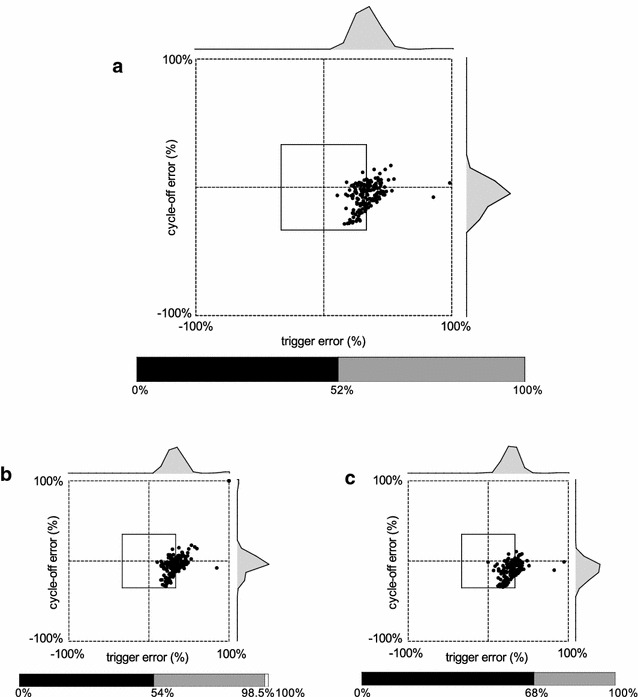
Example of patient–ventilator interaction diagram with good patient–ventilator interaction. Intra-breath patient–ventilator interaction diagrams resulting from automated (**a**) and manual analysis by experts 1 (**b**) and 2 (**c**) are shown. Histograms of trigger and cycle-off errors are shown above and right of interaction diagrams. Stacked bar charts showing the relative distribution of events are depicted under the interaction diagrams. Corresponding NA and Paw tracings are shown in Fig. 3a

**Fig. 5:**
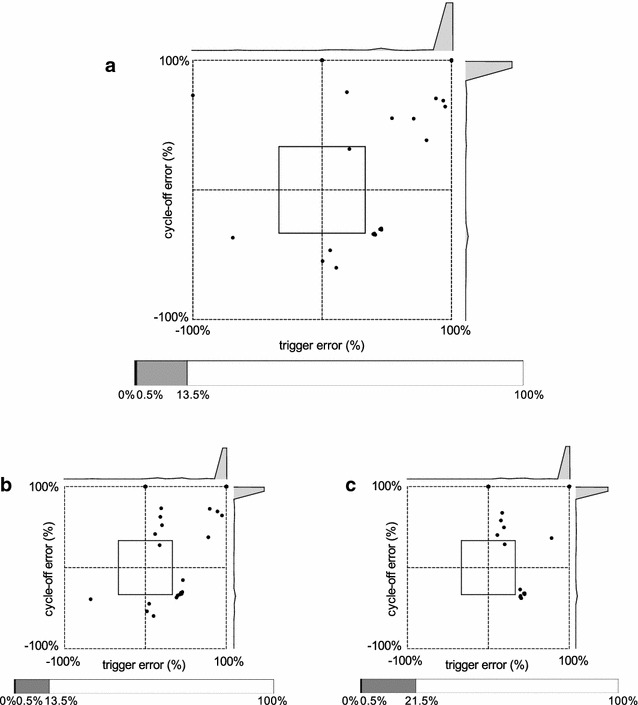
Example of patient–ventilator interaction diagram with poor patient–ventilator interaction. Intra-breath patient–ventilator interaction diagrams resulting from automated (**a**) and manual analysis by experts 1 (**b**) and 2 (**c**) are shown. Histograms of trigger and cycle-off errors are shown above and right of interaction diagrams. Stacked bar charts showing the relative distribution of events are depicted under the interaction diagrams. Corresponding NA and PAW tracings are shown in Fig. 3b

The automated detection algorithm and expert 1 and 2 detected 4366, 4342, 4333 NA or MV breaths, respectively. Based on the dEMG-phase scale_AUTO_, the median rates of synchronous, dyssynchronous and asynchronous breaths as classified by the automated analysis were 11.0% [1.7; 32.7], 49.4% [36.0; 63.9], and 28.7% [6.3; 43.5]. Rates of synchronous, dyssynchronous and asynchronous breaths as classified by the automated analysis are displayed in Fig. 1. Median rates of complete dissociations were 40.4% [18.3; 48.7] for MV without NA, 25.9% [10.2; 48.7] for NA without MV, 4.0% [0; 25.0] multiple MV with NA and 5.8% [0; 15.4] for multiple NA with MV. Rates of complete dissociations as classified by the automated analysis are shown in Fig. 2. Examples of good and poor patient–ventilator interaction with corresponding ventilator pressure and dEMG tracings are shown in Figs. 3, 4 and 5.

### Inter-rater and inter-method agreement

Results for inter-rater and inter-method are displayed in Table 2. The agreement between dEMG-phase scale_MANU_ and dEMG-phase scale_AUTO_ was reflected by an ICC of 1.0 95% CI [0.99–1.0].

**Table 2:** Inter-rater and inter-method agreement

	Inter-rater agreement	Inter-method agreement
Trigger error	0.92 [0.91; 0.92]	0.95 [0.94; 0.95]
Cycle-off error	0.94 [0.94; 0.95]	0.95 [0.95; 0.96]

Inter-rater and method agreements as the intra-class correlation coefficient with a 95% CI

## Discussion

To our knowledge, this is the first study reporting that the interaction between infants and children and the mechanical ventilator can be quantified in a real-time non-invasive manner using transcutaneous electromyographic respiratory muscle recordings. Quantification of patient–ventilator interaction using a modification of a previously described method (dEMG-phase scale) proved to be a feasible and reliable method, reflected by high ICCs for both trigger and cycle-off errors and the dEMG-phase scale. This method may have important implications for both clinical use and research purposes, as it is not restricted to one type of ventilator and it is a non-invasive tool implying that it can be easily implemented in the pediatric population.

To date, measuring the electrical activity of the diaphragm was only feasible using a specifically developed esophageal catheter linked to a specific brand of ventilator. Alternatively, we used surface electrodes with their own limitations. First, when measuring respiratory electrical activity by surface electrodes, also other muscle activity could be measured, a phenomenon known as cross-talk [25]. Reassuring, however, is that we noticed only minimal cross-talk in our study comparable with other studies [17, 26]. Second, electrical interference by machines commonly used in the intensive care unit may interfere with the measured electrical activity [26–29]. We therefore applied a 50 Hz notch filter and were subsequently able to use all data registrations. Third, the use of template subtraction and gating to remove heart activity from the dEMG signal could theoretically interfere with the exact determination of the onset and termination of the neural inspiration. Yet, Hutten et al. used a dEMG signal in which such a filter removed the ECG signal. They found that this filtered signal correlated well with tidal airflow and was fairly robust against time delays [26].

In our previous study, we found that PVA was extremely common in mechanically ventilated children and the predominant type was ineffective triggering [4]. There was some type of asynchrony in one out of every three breaths. Unlike the present study, we had detected PVA by analyzing the ventilator flow and pressure waveforms. Such a method is prone to underreporting the true prevalence of PVA [10]. This is confirmed by the results from the present study, in which we found that only 12.2% (1.9–33.8) of breaths was synchronous. Thus, incorporating dEMG measurements and analyzing the waveforms automatically using the dEMG-phase scale is superior to manual analysis of ventilator waveforms alone. By incorporating the dEMG-phase scale, we were able to improve our definition of PVA [4]. For instance, breaths with relative timing differences > 33% were now classified as dyssynchronous instead of asynchronous, which may explain the difference in occurrence of PVA between this and previous studies. Although the error limits were adopted from Sinderby et al., these limits were arbitrarily chosen and may not be appropriate for defining synchrony, dyssynchrony, and asynchrony in mechanically ventilated children [16]. To determine more accurate inspiration times studies comparing dEMG with the esophageal pressure versus time tracings have to be performed. In addition, in the present study we have used a different brand of ventilator (AVEA, CareFusion, Yorba Linda, CA, USA) than in our previous study (EvitaXL Draeger Medical, Lubeck, Germany). Since a poor patient–ventilator interaction is not only caused by patient but also by ventilator-related factors, it may be surmised that differences in ventilator performance may influence the observed level of asynchrony [30].

Implementing this method to quantify patient–ventilator interaction in the daily evaluation of mechanically ventilated children may be a very promising approach in individually setting the ventilator. For instance, the intra-breath patient–ventilator interaction diagram could be used to adjust the trigger sensitivity and for optimizing cycling criterion. It may be postulated that such guided individual titration may improve patient–ventilator interaction and decrease patient effort, although obviously, this assumption needs to be confirmed in clinical studies. To date, only in observational adults studies a significant association between the level of asynchrony and prolonged duration of mechanical ventilation and mortality has been shown [1, 2]. Pediatric data are lacking. However, a better understanding of patient–ventilator interaction by means of dEMG monitoring may aid in understanding the effects of dys- and asynchrony on patient outcome in ventilated children.

Some limitations of our study need to be discussed. First we used the surface EMG of the diaphragm in the same manner as the EADi signal. To our best knowledge, no correlation studies between the surface EMG and EADi have been performed. Sinderby et al. have shown in a small study population that peak EAdi signals obtained from esophageal catheter were comparable with peak costal surface EMG signal [31]. This manuscript shows that automatic algorithms for transcutaneous electromyographic respiratory muscle recordings to quantify patient–ventilator interaction in mechanically ventilated children can be developed. However, this does not mean surface EMG is equivalent to EADi measurements. More validation studies need to be performed. Secondly, we included patients in the 24 h prior to extubation. The rationale for this was the expectation that patients in the weaning phase are likely to have more interaction with the ventilator. In fact, Emeriaud et al. indeed showed a significant lower diaphragm activity during the acute phase of illness [32]. Last, it should be noted that currently to estimate a patient’s respiratory center output, only dEMG was analyzed. Analyzing both dEMG and EMG of intercostal muscles simultaneously may have an added value in patients characterized by an early trigger error, because the ventilator might be triggered by inspiratory flow generated by intercostal muscle activity. Moreover, it is shown that external intercostal muscles are normally stimulated before the diaphragm as an initial stabilization of the chest wall to make diaphragmatic contraction more efficient [33].

## Conclusions

The transcutaneously measured electrical activation of the diaphragm is a useful signal for evaluating and monitoring patient–ventilator interaction. The dEMG-phase scale was demonstrated to be reproducible and to be an accurate scale to quantify patient–ventilator interaction of mechanically ventilated children. This method may have important implications for both clinical use and research purposes, as it is not restricted to a type of ventilator and it is a non-invasive tool implying that it can be easily implemented in the pediatric population.

The described method could be the first step to determine the effects of patient–ventilator synchrony, dyssynchrony and asynchrony in mechanically ventilated children. Further research is needed to validate cut-off points used in this study. Finally, validation studies are needed to explore the correlation between electrical signals from the diaphragm measured transcutaneously and EADi signals obtained by an esophageal catheter.

## Abbreviations

dEMG: transcuonaeous recording of the electromyographic signals of the diaphragm
ETT: endotracheal tube
FiO_2_: fraction of inspired oxygen
ICC: intra-class correlation coefficient
ICU: intensive care unit
multiple MV with NA: multiple ventilatory pressurizations with one neural activity
multiple NA with MV: multiple neural activities within one ventilatory pressurization
MV without NA: ventilatory pressurization without neural activity
MV: mechanical ventilation
MV_OFF_: end of ventilator pressurization
MV_ON_: beginning of ventilator pressurization
NA without MV: neural activity without ventilatory pressurization
NA_OFF_: termination of neural inspiration
NA_ON_: onset of neural inspiration
PAP: pressure above peep
PC/AC: pressure controlled assist control
PC/SIMV + PSV: pressure controlled/synchronized intermittent mandatory ventilation + pressure support ventilation
PEEP: positive end-expiratory pressure
PICU: pediatric intensive care unit
PIM: Pediatric Index of Mortality
PIP: peak inspiratory pressure
Pmean: mean airway pressure
PRISM: Pediatric RISk of Mortality
PRVC/SIMV + PSV: pressure regulated volume controlled/synchronized intermittent mandatory ventilation + pressure support ventilation
PSV: pressure support ventilation
PVA: patient–ventilator asynchrony
RMS: root mean square
VOXP: Ventilator Open XML Protocol
V_T_: expiratory tidal volume
